# CAR-T Cells in Acute Myeloid Leukemia: Where Do We Stand?

**DOI:** 10.3390/biomedicines12061194

**Published:** 2024-05-28

**Authors:** Daniela Damiani, Mario Tiribelli

**Affiliations:** 1 Division of Hematology and Stem Cell Transplantation, University Hospital, 33100 Udine, Italy; mario.tiribelli@uniud.it; 2 Department of Medicine (DMED), University of Udine, 33100 Udine, Italy

**Keywords:** acute myeloid leukemia, immunotherapy, CAR-T cells, cell targets, prognosis

## Abstract

Despite recent advances, the prognosis of acute myeloid leukemia (AML) remains unsatisfactory due to disease recurrence and the development of resistance to both conventional and novel therapies. Engineered T cells expressing chimeric antigen receptors (CARs) on their cellular surface represent one of the most promising anticancer agents. CAR-T cells are increasingly used in patients with B cell malignancies, with remarkable clinical results despite some immune-related toxicities. However, at present, the role of CAR-T cells in myeloid neoplasms, including AML, is extremely limited, as specific molecular targets for immune cells are generally lacking on AML blasts. Besides the paucity of dispensable targets, as myeloid antigens are often co-expressed on normal hematopoietic stem and progenitor cells with potentially intolerable myeloablation, the AML microenvironment is hostile to T cell proliferation due to inhibitory soluble factors. In addition, the rapidly progressive nature of the disease further complicates the use of CAR-T in AML. This review discusses the current state of CAR-T cell therapy in AML, including the still scanty clinical evidence and the potential approaches to overcome its limitations, including genetic modifications and combinatorial strategies, to make CAR-T cell therapy an effective option for AML patients.

## 1. Introduction

Acute myeloid leukemia (AML) is an aggressive neoplastic disease characterized by an impaired differentiation of hematopoietic stem cells (HSCs) resulting in the accumulation of immature myeloid progenitors and blast cells in the bone marrow (BM) and peripheral blood (PB), a reduced production of mature functional blood cells, and an increased risk of infection and bleeding [1,2,3]. Despite generally attaining a response to first-line treatment, the overall outcome is poor, with a high incidence of relapse and 5-year survival rates around 40–50% in younger patients and less than 15% in older patients [4]. Therapy intensification and allogeneic hematopoietic cell transplantation (HCT) ensure superior survival only in a small fraction of younger patients [5], and those who have never achieved a complete remission (CR) or have experienced early relapse succumb to their disease within a few months [6], underlining the importance of developing novel therapy approaches.

Recent technological progress has significantly improved the knowledge of AML pathogenesis, highlighting the genetic heterogeneity of the disease, which is relevant in determining the response to therapy and, ultimately, the long-term prognosis. Because of the continuous refining of the genetic classification and the recent availability of multiple drugs targeting molecular abnormalities, the current risk stratification and management strategies of AML are mostly based on its genetic and epigenetic signature [7]. However, many lines of evidence prove that AML relapse is favored by the development of a permissive bone marrow microenvironment [8], suggesting a role for immune therapy that might harness an immune response.

In this review, we focused on the potential role of chimeric antigen receptor (CAR)-T cells in the therapy of AML, summarizing the current available data, limitations, mechanisms of resistance and the possible strategies to improve their clinical efficacy.

## 2. CAR-T Cells: Background

Among the strategies to overcome immunosuppression and reactivate the immune response against tumor cells, adoptive cell transfer with CAR-T cells is one of the most innovative. CARs are engineered synthetic receptors able to redirect T lymphocytes to recognize and eliminate cells expressing a specific target antigen in a major histocompatibility (MHC) independent manner [9]. The concept of a chimeric T cell receptor (TCR), combining an antibody-derived variable region (VH/VL) with the TCR to activate the T cell response to antigens, was first reported in 1987 by a Japanese study [10]. In 1989, a similar approach to induce a T cell response in a non-MHC restricted manner was described by immunologists at the Weizmann Institute of Science [11]. They co-transfected a chimeric TCR (cTCR) resulting from the fusion of antibody VH and VL chains with the constant region of the alpha and beta TCR chains in a murine cytotoxic T lymphocyte, obtaining the surface expression of the cTCR and the activation and subsequent killing of the target cells [11]. To bypass the need for a double-chain conventional TCR complex and to improve the low co-transduction efficiency deriving from the need to transfect two separate retroviral vectors, the same group designed a chimeric receptor in which a single-chain variable fragment (scFv) was fused to an intracellular signaling domain from either CD3ζ or FcεRIγ [12]. When expressed in MD.45 T cells, the scFv-receptor (scFvR) independently transduced the T cell activation signals.

Initially referred to as the “T body”, the scFvR represents the prototype of modern CAR. Since then, four/five different generations of CAR-T cells have been developed. At present, a CAR-T cell construct is composed of four main parts: (1) an extracellular antigen-recognition domain, called scFv, able to specifically bind tumor surface antigens, either a tumor-associated antigens (TAAs), the most frequent, or tumor-specific antigens (TSAs). (2) A spacer, also called the hinge region, usually made of either an IgG-based part (such as CH2, CH3, or CH2CH3) or an Ig-based hinge from a naïve T cell molecule (such as CD8 or CD28), to provide flexibility. Together, ScFv and the hinge represent the ectodomain part of CAR. (3) A transmembrane part of CAR, derived from a CD3ζ, CD4, CD8 or CD28 molecule. (4) An intracellular signaling domain, which is mainly a CD3ζ domain. Together, the transmembrane and intracellular domains form the endodomain of CAR. 

In CAR-T cell therapy, CAR transgenes are transferred into previously isolated patient’s naïve T cells and the newly generate CAR-T cells are expanded in vitro and then re-infused into the patients [13,14]. Different transfection methods are used to enforce CAR expression, such as viral vectors, gene editing techniques, mRNA electroporation, or liposomes [15,16,17,18]. Different generations of CAR-T cells have been developed starting form this basic construct to improve their activation and to enhance the CAR-T cell persistence, proliferation and killing activity. The first generation CAR-T cells, comprising only a CD3ζ chain as a signal transmitter from TCR, demonstrated good activity in preclinical murine models but had limited in vivo antitumor action due to their short persistence in the tumor site and to their scanty proliferative capacity [19,20,21,22]. The second generation CAR-T cells were generated by adding a costimulatory domain, such as CD28, 4-1BB (CD137L) or OX40 (CD134) to the CD3ζ domain, thus amplifying signals and inducing a CR in patients with relapsing acute lymphoblastic leukemia (ALL) and in relapsed/refractory (R/R) mature B-cell malignancies [23,24,25,26,27]. 

The unprecedented success of anti-CD19 CAR-T cell therapy in advanced B cell malignancies, with CR rates around 50% and an overall response rate (ORR) in more than 80%, resulted in the approval by the FDA of six anti-CD19 CAR-T cell products since 2017 [28,29,30,31,32,33,34,35]. In multiple myeloma (MM), limited activity has been observed with anti-CD19 CAR-T cells due to the low antigen expression on plasma cells, but encouraging results and good tolerability have been observed in clinical trials with anti-CD138 and anti-BCMA CAR-T cells in R/R MM [36,37]. A recent meta-analysis of 38 reports including a total of 2134 patients with R/R ALL identified 4-1BB costimulatory endodomain, low dose cyclophosphamide leukodepletion and cytologic CR as factors predicting longer survival [38]. Moreover, a retrospective analysis of 809 patients with R/R diffuse large B cell lymphoma (DLBCL) treated with axicabtagen–ciloleucel (using a CD28 costimulatory endodomain in a gammaretroviral vector) demonstrated higher efficacy but also higher toxicity compared to tisagenlecleucel (that uses a 4-1BB costimulatory endodomain in a lentiviral vector) [39]. 

**Figure 1:**
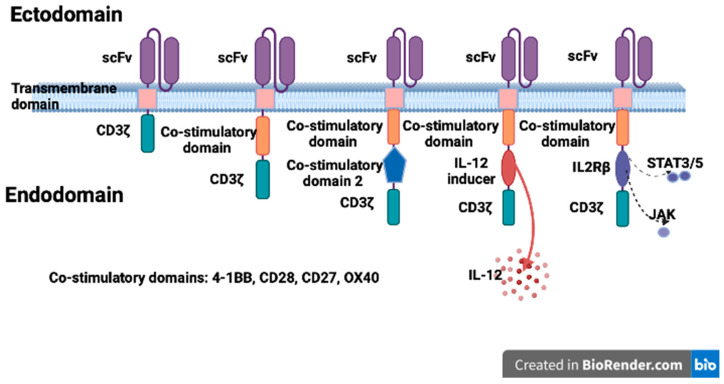
Schematic representation of different CAR structures.

To extend the anti-tumor efficacy a third generation CAR-T cell, comprising two costimulatory endodomains such as CD28-OX40 or CD28-4-1BB, with an improved activation signal, prolonged proliferation and enhanced effector function, has been developed [40]. The CD19-CD3ζ-CD28-4-1BB CAR-T cells induced a high remission rate in R/R ALLs [41]. The fourth generation CAR-T cells, also known as armored or TRUCK CAR-T cells, were engineered from a second-generation CAR endodomain to secrete cytokines, thus improving their function, or equipped with safety switches to regulate the persistence of CAR-T cells (i.e., an inducible caspase 9 suicide switch) and to modulate their on target/off tumor side effects. The fifth generation includes additional expression of a receptor domain, such as the IL2R chain β, to stimulate STAT3/STA5 intracellular pathways. A schematic representation of the different CAR generations is shown in Figure 1.

## 3. CAR-T Cell Therapy in AML

Different from ALL, where anti-CD19 CAR-T cells have demonstrated an impressive response rate, the translation of this approach to AML remains challenging. The lack of specific target antigens, the leukemic clonal heterogeneity, the variable expression in the different phases of the disease, the immunosuppressive role of the leukemic BM microenvironment and the very low mutation frequency in AML compared to solid tumors (which limits the appearance of neo-antigens), all reduce the efficacy and potentially cause unacceptable BM toxicity and off-target adverse events. So, at present, no CAR-T cell therapy has obtained FDA approval for AML treatment.

The first trial reporting the biological activity of CAR-T cells in R/R AML was published by Ritchie et al. in 2013. They used a second-generation CAR targeting the Lewis Y antigen. Lewis Y is a surface glycoprotein expressed on granulocytes, in 50% of AML patients, in the syncytiotrophoblast during embryogenesis and in ovarian cancer, where it is thought to promote tumor invasion and metastasis [42,43]. The trial proved limited efficacy but also very low hematopoietic toxicity, paving the way for the use of CAR-T cells in AML patients [44]. Currently, there are several clinical trials testing CAR-T cells in AML, mostly targeting CLL1, CD123, and CD33, that are all myeloid antigens overexpressed in leukemic blasts but also in their normal stem cell counterpart; however, various other myeloid antigens (e.g., CD64, CD13, CD93, and CD38) as well as repurposed lymphoid antigens (e.g., CD19, CD7, and CD70) are under investigation. The available results, including the very few patients treated to date, are summarized in Table 1.

### 3.1. Potential AML Targets under Clinical Investigation for CAR-T Cell Therapy

#### 3.1.1. CD123

CD123 (IL3R-α) is a membrane protein expressed in most AML blast cells and leukemic stem cells (LSCs) and it is associated with enhanced proliferation, increased cellularity, and a poor prognosis [56,57,58]. CD123 is currently the target for different therapies, including recombinant fusion proteins, neutralizing antibodies, and CAR-T cell therapies [59]. In the field of CAR-T cells, at present CD123 is the most used target with over 20 clinical trials ongoing. As reported in Table 1, Yao et al. treated one patient with relapsed AML with second generation allogeneic CAR-T cells as part of a HCT conditioning regimen, obtaining a CR with incomplete hematologic recovery (CRi), but the patient died from graft versus host disease on day 56 [45]. Cummins et al. enrolled seven R/R AML patients in a phase I trial testing “biodegradable” T cells transiently expressing CD123 CAR. No reduction of CD123 cells was observed, despite the absence of hematologic toxicity and of treatment-related deaths [60]. An early phase pilot study, sponsored by the University of Pennsylvania, investigating an anti-CD123-CD3-4-1BB CAR in five R/R AML patients, was interrupted for lack of efficacy and on target/off tumor adverse events [61]. Budde et al. reported preliminary results employing autologous CD123 CAR-T cells in seven heavily pretreated R/R AML patients with the aim to obtain maximum disease reduction before a second allogeneic HCT, observing a CR in two patients, a morphologic response in one patient and a reduction of blast cells in two patients [62].

#### 3.1.2. CD33

CD33 is a 40 kDa glycoprotein, also known as Siglec3, normally expressed on mature granulocytes and monocytes and with minimal expression on normal HSCs. Approximately 90% of adult AML and 80% of pediatric AML cells express CD33, as well as 9% of LSCs [63]. In preclinical studies in vitro and animal models, CD33-directed CAR-T cells demonstrated high efficacy against AML cells [64], long persistence and prolongation of survival in mice [65,66]. Wang et al. reported that, in the absence of lymphodepletion, anti-CD33 CAR-T cells produced a transient reduction of blast cells, but also grade 4 toxicity [47]. Tambaro et al. at the MD Anderson Cancer Center used autologous CD33 CAR-T cells in three adult R/R AML patients: two of the three patients developed grade 3 toxicity, and all three patients died from leukemia progression [67]. Many other clinical trials with anti-CD33 CAR-T cells, CAR-NK and γδ T cells are currently ongoing.

#### 3.1.3. CLL-1

C-type-lectin-like molecule-1 (CLL-1) is an inhibitory receptor expressed in AML cells and in LCSs of adult and pediatric patients [59]. Second generation CLL1-directed CAR-T cells have been shown to prolong survival in immunodeficient leukemic mice [68,69]. In vivo and in vitro studies with third generation anti-CLL1 CAR-T cells showed high anti leukemia activity and wide production of effector cytokines [69]. Clinical use of anti-CLL1 CAR-T cells has been reported in three pediatric AML patients: all achieved a CR and underwent allogeneic HCT [50]. Jin et al. observed a CR/CRi in 7/10 R/R adult AML patients receiving CLL1-directed CAR-T cells after lymphodepletion with cyclophosphamide and fludarabine; six patients experienced high grade CRS while no patient developed CAR-T cell related encephalopathy syndrome (CRES) [70]. Since CLL1 is highly expressed on normal granulocytes, bridging to allogeneic HCT can be a strategy to rescue long term agranulocytosis due to off target toxicity.

#### 3.1.4. CD38

CD38 is a surface glycoprotein involved in cell adhesion, migration and signal transduction, and is expressed in almost all mature blood cells and in myeloid progenitors, but not in healthy HSCs [71]. Due to its broad expression in MM, T-ALL and AML, CD38 has been recently proposed as a pan-hematologic target for CAR-T cell therapy [72]. In AML, anti-CD38 CAR-T cells were used in six patients relapsing after HCT; four weeks after CAR-T infusion, 4/6 (66.7%) patients achieved a CR/CRi. The cumulative relapse rate at 6 months was 50%. All six patients experienced manageable side effects, and multiparametric flow cytometry (FCM) revealed that the CD38-directed CAR-T cells had cleared the CD38 positive blast cells without off-target effects on normal monocytes or lymphocytes [52].

#### 3.1.5. CD7

CD7 is a glycoprotein essential for T cell and T cell/B cell interactions during early lymphoid development. CD7 is expressed on thymocytes, NK precursors, T-lymphocytes and in about 30% of AML cases, where its high expression level has been associated with more aggressive disease and resistance to conventional therapy [73]. The potential limit is the high CD7 expression on normal lymphocytes, but Gomes-Silva et al. demonstrated that the use of CD7-directed CAR-T cells in CD7 gene-knockout T cells can eliminate AML cells [74]. Cao et al. reported on the use of CD7 autologous CAR-T cells in a young female with R/R AML with a complex karyotype, TP53 deletion, FLT3-ITD mutation and SKAP2–RUNX 1 fusion gene: CD7 CAR-T cells were administered after lymphodepletion with decitabine, fludarabine and cyclophosphamide; 17 days after CAR-T cells infusion, the patient achieved morphologic leukemia-free state (MLFS). No severe organ or CRES toxicity were observed and two months after CAR-T cell infusion the patient underwent an allogeneic HCT, achieving an MRD-negative CR [75]. Hu et al. treated one patient with R/R AML in a phase I trial of anti-CD7 CAR-T cells in hematological malignancies, with a CR 28 days after CAR-T cell infusion; no life-threatening toxicity was observed [76]. Altogether, these data suggest that CD7-directed CAR-T cells can be a suitable therapy for R/R AML.

#### 3.1.6. CD19

CD19 (B-lymphocytes surface antigen B4) is a marker of B cell differentiation with expression restricted to B-lymphocytes. CD19 is crucial for regulating B-cell activation via B-cell receptor-dependent and -independent signaling pathways [77]. Aberrant expression of CD19 has been identified in t(8;21) AML and in 66% of mixed-phenotype acute leukemia [78,79]. Danylesko et al. employed second-generation anti-CD19 CAR-T cells in a patient with t(8;21) AML relapsing after allogeneic HCT. Lymphodepletion was obtained with fludarabine and cyclophosphamide and a persistent clinical and molecular CR was reached on day 28 after CAR-T cell infusion. Grade 3 CRS required inotropic support and tocilizumab [80]. Qu et al. observed similar results in two young patients with R/R AML with t(8;21), suggesting that CD19-directed CAR-T cell therapy is a promising and safe approach to manage R/R t(8;21) AML [55].

#### 3.1.7. CD70

CD70 is a member of the TNF receptor superfamily that plays an important role in immune responses through interactions with CD27 [81]. In AML, CD70 is expressed in many leukemic cells and in LSCs, but not in normal HSCs, making it a promising therapeutic target for AML patients [82,83]. Sauer et al. investigated several anti-CD70 constructs with different hinge regions and costimulatory domains, reporting potent anti-leukemia activity without HSC toxicity in two xenograft models [84]. Wu et al. produced second-generation anti-CD70 CAR-T cells with a 4-1BB costimulatory molecule with high cytotoxic activity and activation ability in a MOLM 13 xenograft mouse model. However, despite promising results in vitro, such CAR-T cells do not completely eliminate leukemia cells in vivo, suggesting the need either to generate a combinatorial CAR construct or to increase CD70 expression on leukemic cells before CAR-T cell exposure [85]. Along this latter line, Riether et al. previously demonstrated that hypomethylating agents (HMAs) increase CD70 expression in LSCs through hypomethylation of the CD70 promoter, enhancing the efficacy of the anti-CD70 antibody cusatuzumab in eliminating LSC in vitro and in vivo [86]. A phase I study of anti-CD70 CAR-T cells for CD70 positive hematologic neoplasms, including AML, is currently recruiting (NCT04662294).

#### 3.1.8. CD44v6

CD44v6 is the splicing variant 6 of CD44, a surface glycoprotein involved in leukocyte activation and malignant transformation. Its expression is restricted to neoplastic cells, where it is required for tumor growth, making it a good candidate for CAR-T cell therapy, also considering that CD44 expression has been associated with a poor prognosis in AML and MM [87]. In an immunocompromised mouse model, Casucci et al. demonstrated that a second generation of T cells targeting CD44v6 mediated potent antitumor effects against primary AML and MM while sparing normal HSCs and CD44v6-expressing keratinocytes [88]. Monocytopenia was observed as the only hematologic toxicity. A phase I/II clinical trial (NCT04097301) in R/R AML and MM with a single dose of anti-CD44v6 CAR-T cells after lymphodepletion with fludarabine and cyclophosphamide was prematurely terminated due to a low recruitment rate; no hematologic response was observed in 2/2 evaluable patients, and in one patient, CD44v6 CAR-T cells were detectable at day 14 and 21. Neutropenia, anemia and pneumonia were observed as off-target toxicities.

#### 3.1.9. FLT3

FLT3 (Fms-like-tyrosine kinase 3) is a cytokine receptor belonging to the receptor tyrosine kinase III. In the hematopoietic system it is expressed in myeloid progenitors, in B-cells, in dendritic cells and in NK cells, and it is involved in stem cell maintenance and differentiation. In AML, FLT3 internal tandem duplication (ITD) is found in around 25% of patients and point mutations in the tyrosine kinase domain (TKD) in about 5–7% [89,90]. The mutated FLT3 kinase activates the PI3K/AKT, JAK/STAT5, and MEK/ERK pathways and promotes leukemia progression [91]. FLT3 mutations have been associated with reduced survival [92]. On this basis, FLT3 is a potential target for CAR-T therapy. In in vitro studies, the binding of a second-generation CAR-T cell with leukemic cells induced the release of IFN-γ and IL-2 [93]. In mice, an inhibition of leukemic cell proliferation with acceptable hematologic toxicity were observed [94]. Karbowski et al. used cynomolgus monkeys to “mimic” the human microenvironment to test tolerability, safety, and dose-dependent efficacy vs. toxicity [95]. At present, some clinical trials of FLT3-directed CAR-T cells are recruiting, but results are not yet available.

#### 3.1.10. NKG2D

Natural killer group 2D (NKG2D) is an activating receptor of T cells and NK cells for recognition of abnormal cells (i.e., leukemic blasts). NKG2D interacts with MHC class I related molecules (the MIC family or ULBP6 family). A broad expression of NKG2D ligands has been found in hematologic malignancies, such as AML and MM, and in solid tumors, but not in normal cells [96,97], thus making NKG2D a potential target for CAR-T cell therapy in AML. Baumeister et al. tested anti-NKG2D CAR-T cells in a first-in-human phase I clinical trial, observing a safe profile but no clinical responses [53]. Driouk et al. increased the efficacy of anti-NKG2D CAR-T cells in vitro by inducing ligand upregulation with histone deacetylase (HDAC) inhibitors [98]. Sallman et al. recently published the results of a multicenter open-label, dose-escalation phase I study of NKG2D-directed CAR-T cells for R/R AML, MDS and MM. They reported a transitory response in 3/12 patients, with two of them later proceeding to allogeneic HCT; seven patients had grade 3–4 treatment-related toxicity across all dose levels, suggesting the need for a combinatorial antigen targeted approach [54].

#### 3.1.11. WT1

Wilms tumor 1 (WT1) is an oncogenic, zinc-finger transcription factor with an important physiologic role in organ development, cell differentiation, proliferation and apoptosis [99]. In AML, WT1 overexpression has been reported in 70% of patients and seems to be correlated with a poor prognosis [100]. Chapuis et al. treated 12 AML patients relapsed after allogeneic HCT with anti-WT1 CAR-T cells; all of the patients responded, with a relapse-free survival of 100% at a median of 44 months, compared to 54% in patients not receiving CAR-T cell therapy. Moreover, they observed long-term persistence of the CAR-T cells [101].

#### 3.1.12. ILT3

ILT3, or LILRB4, is an inhibitor of MHC class I immune activation, and structurally it is like NK cells’ KIR receptors. ILT3 presentation by antigen-presenting cells (APCs) induces CD4+ T helper cell anergy and CD8+ T suppressor cell differentiation. Expression of ILT3 has been observed in monocytic cells and in monocytic/monoblastic AML [102], where its expression may change during disease progression and upon therapeutic pressure [103]. Preclinical in vitro and in vivo studies demonstrated the efficacy of an anti-ILT3 cell model against ILT3+ AML cell lines [104]. An early phase I trial of anti-ILT3 CAR-T cells in R/R M4-M5 AML is currently recruiting (NCT04803929).

#### 3.1.13. Siglec-6

Siglec-6 is an adhesion molecule expressed on mast cells and granulocytes, in mucosal lymphoid cells, and in placental syncytiotrophoblasts. Jetani et al. developed anti-Siglec-6 CAR-T cells with promising killing activity in AML cell lines [105]. A phase I/II clinical study of anti-Siglec-6 CAR-T cells in R/R AML is active in China (NCT05488132).

## 4. Limitations and Challenges in CAR-T Cell Therapy

Many other molecules with variable expression in different AML subsets are under in vitro investigation as potential targets of CAR-T cells. However, at present, none fulfills all of the criteria for being an ideal target for immune therapy. Many are expressed only in some AML subsets, and probably do not justify the costs of large-scale production; others are partially expressed in normal HSCs, increasing the risk of on-target/off-tumor side effects. Moreover, some leukemic antigens may be down modulated under therapeutic pressure, increasing the possibility of antigen escape. The identification of newly formed antigens that result from a mutation associated with or driven by AML itself would represent the preferred target for CAR-T cells [106].

Technological advances, such as whole genome sequencing, and the development of algorithms for epitope prediction, might facilitate the identification of targets potentially suitable for immunotherapies. The Cancer Genome Atlas Research Network conducted a comprehensive study to examine the mutational composition in AML, identifying several recurrent mutations involved in leukemogenesis [107]. However, it must be underlined once more that AML genomes are amongst those with the lowest mutational burden, and very few neo-antigens can be expected [108]. Nonetheless, some neoantigens have been identified, including mutations in the metabolic enzymes IDH1 and IDH2, present in about 20% of AML cases overall [109], to which immunogenicity has been demonstrated [110]. Even mutations of the NPM1 gene, one of the most frequent genetic alterations in AML, have been shown to be immunogenic, inducing CD4+ and CD8+ T cell responses, becoming potential candidate targets for immune therapy. The intracellular expression of these antigens may represent a limitation, but Rafiq et al. generated a CAR construct against WT1, thus demonstrating not only the possibility to extending CAR-T recognition beyond extracellular antigens, increasing the number of potential leukemia-specific targets, but also that these antigens can be harnessed for CAR-T cell therapy [111]. A different source of neoantigens is dysregulated splicing, resulting in neo-isoforms distinct from the wild type counterpart. Adamia et al. used genome-wide alternative splicing screening in a cohort of AML cells, finding that approximately 29% of them expressed differently spliced oncogenes, tumor suppressor proteins, splicing factors, and proteins involved in apoptosis, cell proliferation, and spliceosome assembly; they concluded that aberrant splicing is a common feature in AML, and that the splice variants may provide novel disease markers and potential targets for small molecules or immune therapies [112]. An example of this mechanism is the CD44 isoform, called the CD44v6 variant, discussed above. Splice variants of FLT3 and NOTCH2 have also been reported in over 50% of AML cases, but not in normal hematopoietic progenitors [113].

Antigen loss or downregulation, used by AML blasts to escape immune surveillance, is another cause of limited efficacy of CAR-T cell therapies. Strategies to enhance target antigen expression are needed to improve the CAR-T cell response. Targeting multiple antigens has been proposed as a mechanism to harness CAR-T cell activity. Bispecific CAR-T cells, in which two or more CARs with distinct antigen-recognition domains are used, can result in hypofunction and CAR-T cell exhaustion due to signaling excess and activation-induced death [114,115]. Tandem CAR-T cells, in which the CAR consists of distinct antigen recognizing domains and a single intracellular domain, may enhance therapeutic efficacy [116], but they require CAR optimization to bypass the distance between the CAR and target cells [117]. Mixing different CAR-T cells recognizing individual antigens might overcome this limitation, but at extremely high production costs [118]. However, a trial of therapy with a CD38/CD33/CD56/CD123/CD117/CCD133/CD34/Mucl-directed CAR-T cell is currently recruiting patients at Zhujiang hospital in China (NCT03473457).

Moreover, leukemic cells can induce a permissive microenvironment by upregulating inhibitory ligands that bind to checkpoint receptors on CAR-T cells [119], preventing their function by inducing exhaustion and anergy [120,121]. Despite the success of immune checkpoint blockade in solid tumors, only modest efficacy has been demonstrated in early trials in AML [122,123]. The mechanism of poor efficacy of checkpoint inhibitors is not well understood, but possibly their use in R/R disease or cases with high leukemic burden may be an explanation. However, their combination with CAR-T cells could enhance the response and improve T cell persistence. CAR-T cells have been designed to block PD-1 through secreted single chain variable fragments (scFv), antibodies, shRNA, dominant negative receptors and CRISPR/cas9 mediated knockout [124,125,126]; anti-PD-1 CAR-T cells are under investigation in B cell malignancies and solid tumors. Fan et al. recently demonstrated, both in vitro and in xenograft models, the antileukemic activity of B7-H3 CAR modified-T cells, which prolonged mouse survival without toxicity to normal cells [127]. Lin et al. reported the enhanced cytotoxic effect of anti-CLL1 CAR-T cell therapy in AML cell lines and in blasts from R/R AML patients [49]. Ma et al. described two cases of R/R AML patients, relapsed after HCT and failing multiple salvage therapies including CD38-directed CAR-T cells, that were successfully treated with PD1-silenced anti-CCL1 CAR-T cells: both patients achieved a molecular CRi 28 days after CAR-T cell infusion and maintained continuous remission for 8 and 3 months, respectively [128].

Besides checkpoint inhibitor upregulation, leukemic cells evade immune surveillance by producing immune-modulating enzymes such as arginase II, indoleamine 2,3 dioxygenase and ectonucleotides, leading to the accumulation of immunosuppressive metabolites and suppression of CAR-T cell proliferation and effector function [129]. Strategies to rebalance the leukemia microenvironment by reversing the proinflammatory signals that increase mesenchymal cells (MSC), myeloid-derived suppressor cells (MDSC) and T regulatory cells (Tregs) could counteract leukemia resistance. Anti-CD33 CAR-T cells target CD33 positive blast cells but also MDSC; third-generation CAR-T cells with point mutations in the CD28 endodomain reduce IL2 secretion and consequently Tregs activity [130]. The fourth-generation CAR-T cells, engineered to secrete IL7 and IL15, demonstrated high anti-leukemia activity in preclinical animal models [131,132].

The lack of AML-specific antigens, the co-expression of targets by hematopoietic progenitors and endothelial cells, and the long persistence of CAR-T cells account for the prolonged myeloablation after CAR-T infusion, due to significant on-target/off-tumor toxicity; all of these make CAR-T cells an ideal bridge to allogeneic HCT. Besides pancytopenia, the three principal expressions of CAR-T toxicity are: (a) CRS, a potentially life threatening inflammatory response characterized by fever, hypotension, tachycardia, respiratory distress and multiorgan dysfunction [133,134]; (b) CRES, a CAR-T associated neurological toxicity characterized by confusion, delirium, seizure, and cerebral edema [133,134]; and (c) CAR-associate hemophagocytic lymphohistiocytosis (carHLH), a rare complication characterized by fever, cytopenia, hypertriglyceridemia and high ferritin levels [135].

Another challenge in AML is CAR-T manufacturing, as collection, modification and expansion of autologous T cells is time consuming and complicated, considering the aggressivity of the disease and the different peripheral T cell compositions, influenced by the disease and previous treatment, which can affect CAR-T cell expansion, persistence and efficacy [136].

## 5. Beyond CAR-T Cells

Considering the actual limitations of CAR-T cells, CAR-NK cells may represent a valid alternative. NK cells are a subset of cytotoxic lymphocytes recognizing target cells in the absence of MHC. One major advantage of CAR-NK cells is that autologous cells are not required for their manufacture, and various source can be used, including the NK92 cancer cell line, and thus “ready to use” CAR-NK cells can be manufactured through mass production and infused into patients at any time. A second advantage is the mild CRS and neurotoxicity, probably due to the difference in cytokine release upon cell activation [137]. Moreover, NK cells have multiple mechanisms to target leukemic cells beyond the CAR pathway, including antibody-dependent cell-mediated cytotoxicity (ADCC) and KIR-mediated killing activity. Finally, CAR-NK cells have a limited lifespan, reducing CAR-related toxicity; a potential drawback may be the need for repeated infusions to prolong remission. CD33 and CD123 are the most used targets, but recently CAR-NK cells directed against NKG2D, CD70, CD38, and CLL1 have been generated and proved cytotoxic activity in AML preclinical studies [138,139,140,141]. Many clinical trials of CAR-NK cell therapy are currently recruiting; to date, only two of them have been completed with published results. Tang at al. treated three R/R AML patients with anti-CD33 CAR-NK cells, with low toxicity but progressive disease in all patients [48]. Huang et al. used anti-CD33 CAR-NK cells in ten patients with R/R AML, with a 20% CR rate and low grade CRS [142].

## 6. Conclusions/Perspectives

CAR-T cell therapy in AML has not replicated the enthusiastic results obtained in ALL and B cell lymphomas. Despite several in vitro and animal models proving good efficacy against AML, results in patients are, to date, scarce and disappointing. The variable expression of the most frequent targets and the negative effect of the leukemic microenvironment justify, at least in part, the limited use of CAR-T cells in AML. It must be underlined that AML kinetics are different from that of lymphomas and MM, and the timing of CAR-T can heavily affect disease control. In addition, the manufacture of CAR-T cells itself may be a challenge in patients with active disease due to the detrimental effects of AML chemotherapies toward T cells. All of the in vivo studies have been performed in R/R disease but it is arguable that the use of CAR-T cell therapy in an earlier phase of disease may control minimal residual disease (MRD) and, possibly, increase the success rate. Lastly, AML is a dynamic condition and a global immune approach, combining cellular therapies and targeted small molecules, could represent a way to enhance the response rates in this heinous disease.

In conclusion, we think that CAR-T cell therapy also has an exciting outlook in myeloid neoplasms, though its efficacy and safety still require well-designed clinical trials to define the optimal setting in which this new approach is most effective. In our opinion, this is mandatory for a correct allocation of the limited financial resources, considering the estimated increase in costs for CAR-T cell therapy, for the management of specific toxicities of cellular therapies, and in view of the possible replacement of “conventional” therapies, including allogeneic transplant, with CAR-T cells. This is utterly true in AML, a heterogeneous disease with multiple mechanisms of immune escape, where many questions still need an answer. For instance, which is the most appropriate antigen and what is the most appropriate timing of CAR-T in the therapeutic project? Should CAR-T cells be used at disease relapse, or in case of measurable minimal residual disease, as a bridge to transplant or as a maintenance therapy alternative to stem cell transplant? A second point still in need of definition is about the timing of autologous T cell collection; considering the impact of chemotherapies on T cell subpopulations, an early collection may preserve their number and function. A further issue is the mitigation of CAR-T cell on target/off tumor toxicity, mainly prolonged cytopenia, due to a lack of specificity of antigen expression in AML cells. It should be investigated whether it is more effective to target neoantigens, rare and often with low expression, or to employ a tandem/multitarget approach to better discriminate leukemic and normal hematopoietic cells; furthermore, we should test in clinical trials if is preferable to administer multiple infusions of low CAR-T cell doses, or to limit CAR-T persistence by engineering “off switches” or “suicide genes” into the CAR construct.

A prompt but exhaustive response to these questions will contribute to better defining CAR-T cell efficacy in the setting of AML, limiting the risks of regulatory non-approval and, in case of the entrance of this novel therapy into the clinical scenario, aiding in the identification of the correct patient, thus preventing an economic collapse of health systems.

## Figures and Tables

**Table 1:** Summary of published data on CAR-T in AML. Studies are grouped by target molecule and by year of publication.

	Target Antigen		Transduction Mechanism	Costimulatory Domain	CAR-T Cells Source	AML Status/ Patients N°	Prior HCT	Outcome	Toxicity
Ritchie et al., 2013 [44]	LeY Ag	Phase I	Lentiviral	CD28-CD3ζ	auto	Ref: 3 Rel: 1	no	ORR: 2 (50%) CR: 1 (25%)	0
Yao et al., 2019 [45]	CD123	Case report	Retroviral	41BB	allo	Rel: 1	yes	ORR: 1 (100%) CR: 1 (100%)	0
Wermke et al., 2021 [46]	CD123	Phase I	NA	CD28	auto	Rel/Ref: 3	yes (2 pts)	ORR: 3 (100%) CR: 2 (67%)	CRS: 2 (67%, I) CRES: 0
Wang et al., 2015 [47]	CD33	Phase I	Lentiviral	41BB-CD3ζ	auto	Rel/Ref: 1	no	ORR: 1 (100%)	CRS 1 (100%, IV)
Tang et al., 2018 [48]	CD33	Phase I	Lentiviral	CD28-41BB	NK92 cells	Rel: 3	yes (1 pt)	ORR: 2 (67%) CR: 1 (33%)	CRS: 2 (67%, I–II)
Lin et al., 2021 [49]	CLL-1	Phase I	Lentiviral	CD28-CD3ζ	auto-allo	Ref: 1 Rel: 9	no	NA	NA
Zhang et al., 2021 [50]	CLL-1	Phase I/II	Lentiviral	CD28-CD3ζ-CD27	auto	Rel/Ref: 4	no	ORR: 3 (75%) CR: (75%)	CRS: 3 (75%, I–II) CRES 1 (25%)
Liu et al., 2020 [51]	CLL1/CD33	Phase I	NA	NA	auto-allo	Rel/Ref: 9	NA	ORR: 7 (78%) CR: 7 (78%)	CRS: 8 (89%) 3I, 3II, 2II CRES: 4 (44%)
Cui et al., 2021 [52]	CD38	Phase I	NA	41BB-CD3ζ	auto-allo	Rel/Ref: 6	yes	ORR: 4 (67%) CR: 4 (67%)	CRS 5 (83% I–II) CRS 1 (17%, III)
Baumeister et al., 2019 [53]	NKG2DL		Retroviral	CD3ζ-Dap10	auto	Ref: 4 Rel: 3	NA	ORR: 0	0
Sallman et al., 2023 [54]	NKG2DL		Retroviral	CD3ζ	auto	Rel/Ref: 1	yes	ORR: 1 (100%) CR: 1 (100%)	0
Qu et al., 2019 [55]	CD19		NA	NA	auto-allo	Ref: 1 Rel: 1	yes (1 pt)	ORR: 2 (100%) CR: 2 (10%)	2 (100%, I–IV)

Ref: refractory; Rel: relapsed; ORR: overall response rate; CR 1: first complete remission; CR 2: second complete remission.

## Data Availability

No new data were created or analyzed in this study. Data sharing is not applicable to this article.
